# Optimal composition and position of histidine-containing tags improves biodistribution of ^99m^Tc-labeled DARPin G3

**DOI:** 10.1038/s41598-019-45795-8

**Published:** 2019-06-28

**Authors:** Anzhelika Vorobyeva, Alexey Schulga, Elena Konovalova, Rezan Güler, John Löfblom, Mattias Sandström, Javad Garousi, Vladimir Chernov, Olga Bragina, Anna Orlova, Vladimir Tolmachev, Sergey M. Deyev

**Affiliations:** 1 Department of Immunology, Genetics and Pathology, Uppsala University, Uppsala, Sweden; 2 Molecular Immunology Laboratory, Shemyakin & Ovchinnikov Institute of Bioorganic Chemistry, Russian Academy of Sciences, Moscow, Russia; 3 Department of Protein Science, School of Engineering Sciences in Chemistry, Biotechnology and Health, KTH Royal Institute of Technology, Stockholm, Sweden; 4 Nuclear Medicine and PET, Department of Surgical Sciences, Uppsala University, Uppsala, Sweden; 5 Nuclear Medicine Department, Cancer Research Institute, Tomsk National Research Medical Center Russian Academy of Sciences, Tomsk, Russia; 6 Department of Medicinal Chemistry, Uppsala University, Uppsala, Sweden; 7 Science for Life Laboratory, Uppsala University, Uppsala, Sweden; 8 National Research Tomsk Polytechnic University, Tomsk, Russia; 9 Bio-Nanophotonic Lab, Institute of Engineering Physics for Biomedicine (PhysBio), National Research Nuclear University “MEPhI”, Moscow, Russia

**Keywords:** Predictive markers, Cancer

## Abstract

Radionuclide molecular imaging of HER2 expression in disseminated cancer enables stratification of patients for HER2-targeted therapies. DARPin G3, a small (14 kDa) engineered scaffold protein, is a promising probe for imaging of HER2. We hypothesized that position (C- or N-terminus) and composition (hexahistidine or (HE)_3_) of histidine-containing tags would influence the biodistribution of [^99m^Tc]Tc(CO)_3_-labeled DARPin G3. To test the hypothesis, G3 variants containing tags at N-terminus (H_6_-G3 and (HE)_3_-G3) or at C-terminus (G3-H_6_ and G3-(HE)_3_) were labeled with [^99m^Tc]Tc(CO)_3_. Labeling yield, label stability, specificity and affinity of the binding to HER2, biodistribution and tumor targeting properties of these variants were compared side-by-side. There was no substantial influence of position and composition of the tags on binding of [^99m^Tc]Tc(CO)_3_-labeled variants to HER2. The specificity of HER2 targeting *in vivo* was confirmed. The tumor uptake in BALB/c nu/nu mice bearing SKOV3 xenografts was similar for all variants. On the opposite, there was a strong influence of the tags on uptake in normal tissues. The tumor-to-liver ratio for [^99m^Tc]Tc(CO)_3_-(HE)_3_-G3 was three-fold higher compared to the hexahistidine-tag containing variants. Overall, [^99m^Tc]Tc(CO)_3_-(HE)_3_-G3 variant provided the highest tumor-to-lung, tumor-to-liver, tumor-to-bone and tumor-to-muscle ratios, which should improve sensitivity of HER2 imaging in these common metastatic sites.

## Introduction

Current and further progress in efficient treatment of disseminated cancer is often linked with molecular recognition of receptors, which are aberrantly expressed in malignant cells and maintain such hallmarks of cancer as sustained proliferative signaling, active invasiveness, angiogenesis induction, and apoptosis resistance. These receptors can be used as molecular targets for signaling inhibition or delivery of cytotoxic drugs. One such target is the human epidermal growth factor receptor (HER2). Treatment of patients with HER2-positive breast and advanced gastric or gastro-esophageal junction cancers with specific antibodies or tyrosine kinase inhibitors provides significant survival of patients^1,2^. In both cases, high HER2 expression is a predictive biomarker for response to therapy and patients are routinely screened by analysis of biopsy samples. However, an appreciable inter- and intratumoral heterogeneity^3^ of HER2 expression makes this method unreliable. Radionuclide imaging of HER2 is considered a promising way to overcome the problem of heterogeneity of HER2 expression and has the potential to improve precision of cancer treatments^4^. It has been demonstrated in clinical trials that imaging of HER2 expression in tumors using radiolabeled monoclonal anti-HER2 antibody trastuzumab can predict response to treatment with trastuzumab^5^ and the antibody-drug conjugate trastuzumab-DM1^6^.

Although the use of radiolabeled monoclonal antibodies is straightforward, it is associated with long time (4–6 days) between injection and imaging^7^, and with a risk of false-positive findings due to non-target-specific accumulation in tumors because of the “enhanced permeability and retention” (EPR) effect^8^. Reduction in size of a protein-based imaging probe increases imaging contrast, reduces optimal time between injection and imaging, and minimizes the EPR effect^7,9^. Therefore, appreciable efforts are invested in development of imaging probes that are smaller than immunoglobulins^10^.

**Figure 1:**
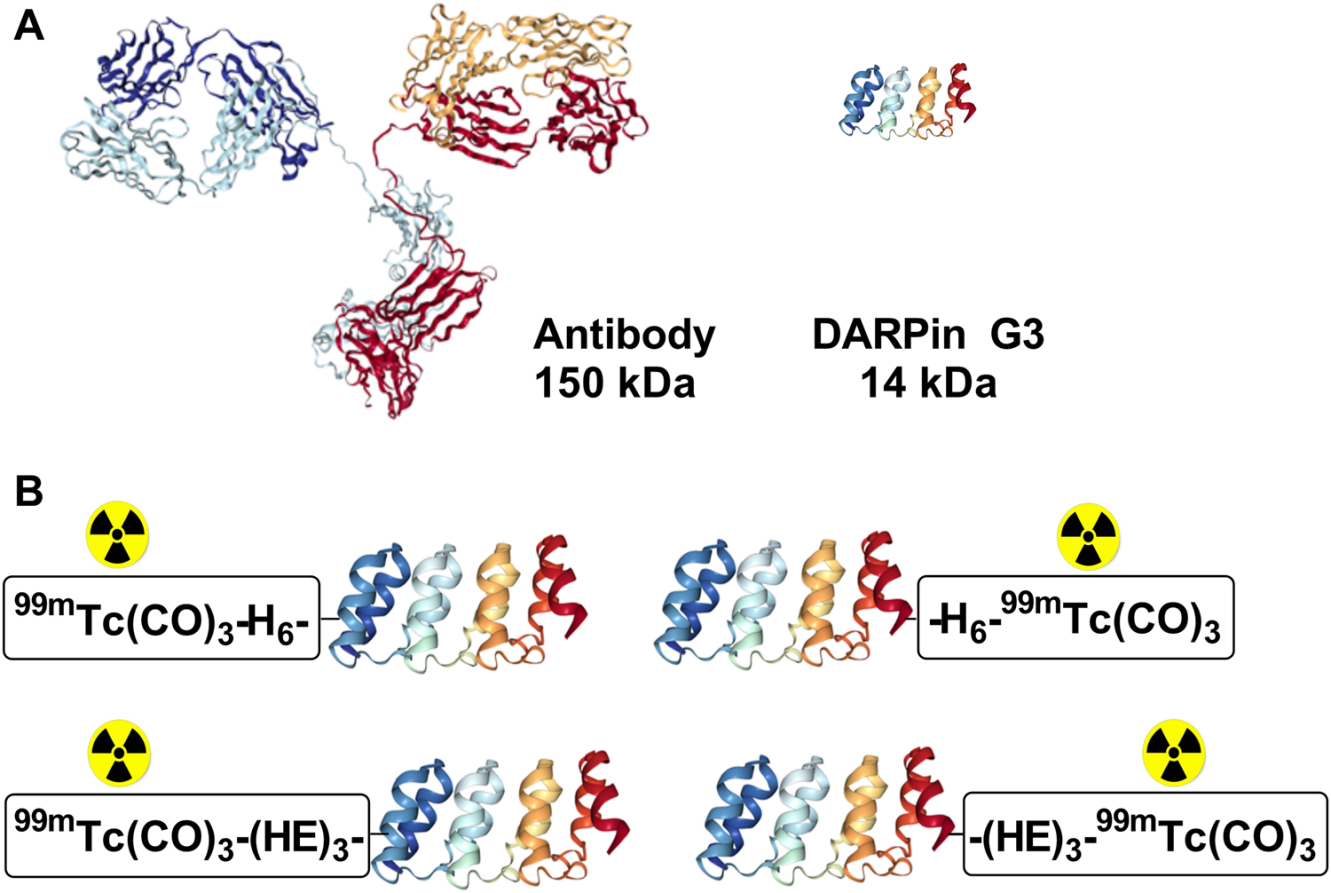
Schematic illustration of proteins used as targeting vehicles for molecular imaging. (**A**) Structures of an IgG monoclonal antibody sized relative to a DARPin G3. (**B**) Four DARPin G3 variants having histidine-containing tags at N-terminus or C-terminus were used in this study. Illustrations were taken from Research Collaboratory for Structural Bioinformatics Protein Data Bank: 1IGT (antibody), 2JAB (DARPin).

One of the possible approaches for the development of small targeting protein probes for imaging is the use of engineered scaffold proteins (ESP)^11,12^. ESPs have variable amino acids incorporated in a structurally rigid core (scaffold). Randomization of variable amino acids enables selection of binders to different molecular target, and the presence of the core minimizes entropy penalty and ensures high binding affinity. One promising class of ESP for molecular imaging are designed ankyrin repeat proteins (DARPins). The DARPin scaffold is built from three or four 33-amino acid-repeats containing a β-turn and two anti-parallel α-helices^13^ (Fig. 1A). DARPins typically have high thermodynamic stability against thermal and chemical denaturation, and high solubility^13^. Another potential advantage of DARPins is the highly efficient production in prokaryotic hosts, which reduces manufacturing costs^13^. High-affinity DARPin-based binders to different molecular targets have been selected for applications in, for example, basic biology research, biosensors, and tumor targeting. Particularly, selection of two DARPins with high affinity to HER2, 9_29 (3.8 nM)^14^ and G3 (0.09 nM)^15^, has been reported. Feasibility of radionuclide targeting of HER2 using G3 having a hexahistidine tag at N-terminus (H_6_-G3) and labeled with [^99m^Tc]Tc(CO)_3_ has been demonstrated^15^. However, [^99m^Tc]Tc(CO)_3_-H_6_-G3 provided a quite low tumor-to-liver ratio (1.1) at 4 h after injection. This would prevent imaging of hepatic metastases, which are frequent in many cancers, including breast carcinoma. Tumor-to-liver ratio for [^99m^Tc]Tc(CO)_3_-H_6_-9_29 DARPin was even lower, 0.11 ± 0.02 at 6 h after injection^16^. It is highly likely that the elevated hepatic uptake was associated with the unfavorable combination of the hexahistidine tag^17,18^ and high lipophilicity of [^99m^Tc]Tc(CO)_3_. The use of the more hydrophilic chelator DOTA with the radionuclide ^111^In and removal of the hexahistidine tag appreciably reduced the hepatic uptake of [^111^In]In-DOTA-G3 and increased the tumor-to-liver ratio up to 12 ± 2^19^.

Another approach to increase the tumor-to-liver ratio for radiolabeled DARPins was based on the use of non-residualizing labels, i.e. labels that “leak” from tumors after internalization and lysosomal degradation^20^. Usually, the use of residualizing labels is necessary for rapidly internalized peptides^21^, although such labels are also trapped in normal tissues and retained there. It was found that internalization of G3 DARPin after binding to cancer cells was slow^22^. Thus, the use of a residualizing radiometal label is not mandatory. In fact, the use of non-residualizing radioiodine label did not cause reduction of tumor uptake of G3 in comparison with the use of the residualizing [^99m^Tc]Tc(CO)_3_ label but resulted in a substantial reduction of hepatic uptake, providing excellent tumor-to-liver ratio, 11 ± 2 at 6 h after injection^22^. Furthermore, direct electrophilic radioiodination was found to be a better labeling approach for G3 compared to indirect radioiodination using HPEM^23^.

A high imaging contrast (i.e. high tumor-to-organ ratios) is the primary goal in the development of imaging probes. Nonetheless, imaging properties of a radionuclide label and economic aspects should also be taken into account to facilitate rapid clinical translation. Indium-111 (t_1/2_ = 2.8 d) provided good tumor-to-organ ratios for G3 DARPin in animal studies. However, this nuclide emits an abundant gamma quantum with the energy of 245 keV. Imaging using ^111^In requires the use of a medium energy general purpose collimator, which reduces both resolution and sensitivity of imaging compared to the low energy high resolution collimator suitable for ^123^I and ^99m^Tc. Besides, this nuclide is quite expensive. The use of ^123^I (t_1/2_ = 13.7 h) for single photon computed tomography (SPECT) imaging or ^124^I (t_1/2_ = 4.18 d), for positron emission tomography (PET) imaging would provide appreciably better resolution and sensitivity compared with the use of ^111^In, though, the costs of the radionuclides would be high as well. The generator-produced ^99m^Tc (t_1/2_ = 6 h) is by orders of magnitude cheaper compared to iodine isotopes. The cost, in combination with excellent availability and favorable dosimetry, makes it a highly attractive label for SPECT applications. Therefore, development of a technetium-based label with optimal biodistribution properties for DARPins is desirable.

Studies on affibody molecules have showed that substitution of a hexahistidine tag by a histidine-glutamate-histidine-glutamate-histidine-glutamate (HEHEHE or (HE)_3_) tag permitted both immobilized metal ion affinity chromatography (IMAC) purification and site-specific labeling with [^99m^Tc]Tc(CO)_3_^24^. At the same time, the use of this tag reduced hepatic uptake of [^99m^Tc]Tc(CO)_3_-labeled anti-HER2 affibody molecules more than 10-fold compared with counterpart molecules containing hexahistidine tag^24^. Interestingly, the effect of a histidine-containing tag on hepatic uptake of affibody molecules was dependent on its position in the protein and the effect was much more pronounced in the case of N-terminal placement compared with the placement at C-terminus^25^. However, the influence of the tag on biodistribution is different for various types of ESPs. For example, the effect of a (HE)_3_-tag on biodistribution of another ESP, ADAPT, was minor^26^.

The goal of this study was to test the hypothesis that position (C- or N-terminus) and composition (hexahistidine or (HE)_3_) of histidine-containing tags would influence the biodistribution of [^99m^Tc]Tc(CO)_3_-labeled anti-HER2 DARPin G3. To test the hypothesis, G3 variants containing tags at N-terminus (H_6_-G3 and (HE)_3_-G3) or C-terminus (G3-H_6_ and G3-(HE)_3_) were labeled with [^99m^Tc]Tc(CO)_3_ (Fig. 1B). Binding to HER2 *in vitro*, biodistribution and tumor targeting properties of these variants were studied side-by-side.

## Results

### Labeling and stability

The identity of non-labeled DARPin G3 variants was confirmed using mass spectrometry analysis (Supplementary Fig. 1). All G3 variants were successfully labeled with tricarbonyl technetium. Incubation of G3 variants with tricarbonyl technetium complex for 60 min at 60 °C provided radiochemical yield over 50% (Table 1). Purification using NAP-5 size-exclusion columns provided radiochemical purities over 97%. The radiocolloid content was in the range of 0.6–1.6%. Radio-HPLC analysis of ^99m^Tc-labeled G3 variants after purification showed a single peak for all proteins (Supplementary Fig. 2).

**Table 1:** Radiochemical yield, isolated yield, radiochemical purity and maximum apparent specific activity of ^99m^Tc-labeled G3 variants. Experiments were performed in duplicates.

Conjugate	Radiochemical yield, %*	Isolated yield, %*	Radiochemical purity, %	Maximum specific activity, MBq/μg (GBq/μmol)
[^99m^Tc]Tc(CO)_3_-H_6_-G3	87 ± 6	72 ± 1	99 ± 0	2.8 (40.1)
[^99m^Tc]Tc(CO)_3_-G3-H_6_	58 ± 4	50 ± 1	99 ± 1	3.0 (42.9)
[^99m^Tc]Tc(CO)_3_-(HE)_3_-G3	72 ± 0	68 ± 2	99 ± 0	3.1 (44.4)
[^99m^Tc]Tc(CO)_3_-G3-(HE)_3_	66 ± 5	57 ± 4	98 ± 1	3.4 (48.6)

*Radiochemical yield is based on iTLC analysis of the crude product;

Isolated yield is defined as a percentage of activity in the high molecular weight fraction after size-exclusion chromatography purification in total activity (leftover in the reaction vial, activity in a column, and activity in the high molecular weight fraction).

All radiolabeled G3 variants demonstrated high stability after incubation with 5000-fold molar excess of histidine up to 3 h (Table 2).

**Table 2:** *In vitro* stability of ^99m^Tc-labeled G3 variants. Samples were incubated with 5000-fold molar excess of histidine or in PBS. Experiments were performed in duplicates.

Conjugate	1 h		3 h	
	PBS	5000x histidine	PBS	5000x histidine
[^99m^Tc]Tc(CO)_3_-H_6_-G3	99 ± 0	99 ± 0	98 ± 0	99 ± 0
[^99m^Tc]Tc(CO)_3_-G3-H_6_	99 ± 0	99 ± 0	99 ± 0	99 ± 0
[^99m^Tc]Tc(CO)_3_-(HE)_3_-G3	99 ± 0	98 ± 0	98 ± 0	97 ± 1
[^99m^Tc]Tc(CO)_3_-G3-(HE)_3_	98 ± 0	98 ± 0	97 ± 0	97 ± 0

### *In vitro* binding specificity and processing by cells

The binding of all radiolabeled G3 variants to cells with high (SKOV3, BT474) and low (DU145) HER2 expression was significantly (p < 0.01) reduced in the presence of a large excess of the corresponding non-labeled DARPin G3 (Fig. 1). The results of the binding specificity experiment showed that the binding was HER2-mediated and radiolabeling did not have a negative influence on binding specificity.

The binding kinetics of radiolabeled G3 variants to living SKOV3 cells was determined using LigandTracer. The binding and dissociation data were best fitted to the one-to-two Langmuir binding model. As it was previously observed for [^99m^Tc]Tc(CO)_3_]-G3-H_6_^22^, all variants had a high affinity interaction in the picomolar range and a low affinity one in the nanomolar range (Table 3).

**Table 3:** Equilibrium dissociation constants (K_*D*_) for the interactions between [^99m^Tc]Tc-labeled G3 variants and HER2-expressing SKOV3 cells. Experiments were performed in duplicates.

Conjugate	K_*D1*_ (pM)	K_*D2*_ (nM)
[^99m^Tc]Tc(CO)_3_-H_6_-G3	90 ± 5	3.3 ± 0.2
[^99m^Tc]Tc(CO)_3_-G3-H_6_	118 ± 38	2.5 ± 1.5
[^99m^Tc]Tc(CO)_3_-(HE)_3_-G3	96 ± 3	1.2 ± 0.1
[^99m^Tc]Tc(CO)_3_-G3-(HE)_3_	89 ± 11	1.8 ± 0.5

Cellular processing of ^99m^Tc-labeled G3 variants by HER2-expressing SKOV3 cells during continuous incubation is presented in Fig. 3. All variants showed a similar pattern of processing with rapid binding to cells and relatively slow internalization. However, some differences between constructs with H_6_ and (HE)_3_ were observed. There was significant (p < 0.005) increase of cell-associated activity between 6 and 24 h for hexahistidine-tag-containing DARPins, but not for the (HE)_3_-containing ones. The internalized fraction at 24 h was higher (significant difference in ANOVA analysis with Bonferroni correction for multiple comparisons) for [^99m^Tc]Tc(CO)_3_-H_6_-G3 and [^99m^Tc]Tc(CO)_3_-G3-H_6_ (37.0 ± 1.5% and 33.2 ± 1.5%, respectively) than for [^99m^Tc]Tc(CO)_3_-(HE)_3_-G3 and [^99m^Tc]Tc(CO)_3_-G3-(HE)_3_ (26.7 ± 0.7% and 25.5 ± 1.5%, respectively). However, the difference was not significant at earlier time points.

### Biodistribution studies

The specificity of HER2-targeting by ^99m^Tc-labeled G3 variants was confirmed in BALB/c nun/nu mice *in vivo*. The tumor uptake in HER2-negative Ramos xenografts was significantly (p < 0.05, unpaired t test) lower than in HER2-positive SKOV3 xenografts (Fig. 4).

Side-by-side comparison of biodistribution of four radiolabeled G3 variants in BALB/c nu/nu mice bearing SKOV3 xenografts is shown in Table 4. All four variants provided similar tumor uptake (7–9%ID/g) at 4 h pi (p > 0.05, one-way ANOVA test) and had good retention in tumors up to 24 h pi with no significant (p > 0.05, one-way ANOVA test) difference between variants, except for a minor decrease for [^99m^Tc]Tc(CO)_3_-G3-(HE)_3_. All variants demonstrated predominantly renal clearance. The liver uptake of [^99m^Tc]Tc(CO)_3_-(HE)_3_-G3 was three-fold lower than of [^99m^Tc]Tc(CO)_3_-H_6_-G3 and about two-fold lower than of [^99m^Tc]Tc(CO)_3_-G3-H_6_ or of [^99m^Tc]Tc(CO)_3_-G3-(HE)_3_. Additionally, the G3 variant with N-terminal (HE)_3_-tag had the lowest uptake of all four variants in lungs, spleen, and muscle at 4 h pi and in liver, spleen and stomach at 24 h pi (p < 0.05, one-way ANOVA test). As a result, it provided the highest tumor-to-organ ratios of all four variants for salivary glands, liver, spleen, stomach and muscles at both 4 h and 24 h pi (p < 0.05, one-way ANOVA test) (Table 5).

**Table 4:** Comparative biodistribution of ^99m^Tc-labeled G3 variants in BALB/C nu/nu mice bearing SKOV3 xenografts at 4 and 24 h pi.

	[^99m^Tc]Tc(CO)_3_-H_6_-G3	[^99m^Tc]Tc(CO)_3_-G3-H_6_	[^99m^Tc]Tc(CO)_3_-(HE)_3_-G3	[^99m^Tc]Tc(CO)_3_-G3-(HE)_3_
**4** **h**				
Blood	0.19 ± 0.06^c^	0.14 ± 0.03^e^	0.18 ± 0.03^f^	0.36 ± 0.07
Salivary glands	1.9 ± 0.2^c^	1.5 ± 0.2	0.7 ± 0.2^b,d^	1. 1 ± 0.3
Lung	0.5 ± 0.1^a^	0.7 ± 0.1	0.34 ± 0.02^b,d,f^	0.6 ± 0.1
Liver	5.5 ± 0.3^c^	4.2 ± 0.9	1.8 ± 0.1^b,d,f^	3.1 ± 0.7
Spleen	0.9 ± 0.1^a^	1.1 ± 0.1	0.47 ± 0.04^b,d,f^	1.0 ± 0.1
Stomach	0.8 ± 0.1	0.8 ± 0.2	0.4 ± 0.1^b,d^	0.7 ± 0.1
Kidney	297 ± 16^a,c^	220 ± 30	237 ± 30	211 ± 41
Tumor	9 ± 3	8 ± 2	9 ± 1	7 ± 1
Muscle	0.21 ± 0.03	0.3 ± 0.1	0.11 ± 0.01^b,d,f^	0.23 ± 0.04
Bone	0.9 ± 0.1	0.9 ± 0.3	0.4 ± 0.1^b,d^	0.7 ± 0.2
**24** **h**				
Blood	0.07 ± 0.01^c^	0.08 ± 0.01^e^	0.09 ± 0.01^f^	0.15 ± 0.01
Salivary glands	1.0 ± 0.3	1.5 ± 0.2^e^	0.4 ± 0.1^d^	0.8 ± 0.2
Lung	0.3 ± 0.1	0.7 ± 0.1	0.3 ± 0.1	0.4 ± 0.1
Liver	3.6 ± 0.4^a,c^	4.6 ± 0.4	1.2 ± 0.2^b,d,f^	2.6 ± 0.1
Spleen	0.9 ± 0.4	1.1 ± 0.1	0.4 ± 0.1^b,d,f^	0.9 ± 0.1
Stomach	0.4 ± 0.1	0.9 ± 0.1	0.22 ± 0.04^b,d,f^	0.40 ± 0.03
Kidney	177 ± 25	212 ± 31	134 ± 16^b^	144 ± 7
Tumor	8 ± 2^c^	9 ± 1	7 ± 2	5 ± 1
Muscle	0.16 ± 0.02	0.31 ± 0.04	0.08 ± 0.02^d,f^	0.20 ± 0.04
Bone	0.5 ± 0.1^c^	1.0 ± 0.3	0.3 ± 0.1^d,f^	0.8 ± 0.1

Data are presented as mean %ID/g values with SD from four mice. One-way ANOVA with Bonferroni’s multiple comparisons test was performed to find significant differences. ^a^Significant difference between [^99m^Tc]Tc(CO)_3_-H_6_-G3 and [^99m^Tc]Tc(CO)_3_-G3-H_6_. ^b^Significant difference between [^99m^Tc]Tc(CO)_3_-H_6_-G3 and [^99m^Tc]Tc(CO)_3_-(HE)_3_-G3. ^c^Significant difference between [^99m^Tc]Tc(CO)_3_-H_6_-G3 and [^99m^Tc]Tc(CO)_3_-G3-(HE)_3_. ^d^Significant difference between [^99m^Tc]Tc(CO)_3_-G3-H_6_ and [^99m^Tc]Tc(CO)_3_-(HE)_3_-G3. ^e^Significant difference between [^99m^Tc]Tc(CO)_3_-G3-H_6_ and [^99m^Tc]Tc(CO)_3_-G3-(HE)_3_. ^f^Significant difference between [^99m^Tc]Tc(CO)_3_-(HE)_3_-G3 and [^99m^Tc]Tc(CO)_3_-G3-(HE)_3_.

**Table 5:** Tumor-to-organ ratios for ^99m^Tc-labeled G3 variants in BALB/C nu/nu mice bearing SKOV3 xenografts at 4 and 24 h pi.

	[^99m^Tc]Tc(CO)_3_-H_6_-G3	[^99m^Tc]Tc(CO)_3_-G3-H_6_	[^99m^Tc]Tc(CO)_3_-(HE)_3_-G3	[^99m^Tc]Tc(CO)_3_-G3-(HE)_3_
**4** **h**				
Blood	46 ± 20^c^	54 ± 7^e^	49 ± 10^f^	19 ± 2
Salivary glands	5 ± 2	5 ± 2	13 ± 4^b,d,f^	6 ± 1
Lung	18 ± 7	11 ± 3	26 ± 3^d,f^	12 ± 2
Liver	1.6 ± 0.7	1.8 ± 0.3	4.9 ± 0.3^b,d,f^	2.2 ± 0.3
Spleen	10 ± 4	7 ± 2	19 ± 2^b,d,f^	7 ± 1
Stomach	12 ± 5	9 ± 2	23 ± 6^b,d,f^	10.2 ± 0.2
Kidney	0.03 ± 0.01	0.04 ± 0.01	0.037 ± 0.004	0.03 ± 0.01
Muscle	42 ± 20	26 ± 6	80 ± 5^b,d,f^	29 ± 4
Bone	10 ± 4	9 ± 3	25 ± 4^b,d,f^	10 ± 2
**24** **h**				
Blood	110 ± 12^a,c^	83 ± 11^e^	80 ± 6^b,f^	32 ± 7
Salivary glands	8 ± 1	4 ± 2	16 ± 3^b,d,f^	6 ± 1
Lung	25 ± 5^a,c^	17 ± 3	28 ± 2^d,f^	12 ± 2
Liver	2.3 ± 0.6	2.3 ± 0.6	5.9 ± 0.9^b,d,f^	1.8 ± 0.4
Spleen	10 ± 3	8 ± 1	20 ± 3^b,d,f^	6 ± 2
Stomach	21 ± 4	12 ± 3	32 ± 7^b,d,f^	12 ± 3
Kidney	0.045 ± 0.002	0.04 ± 0.01	0.05 ± 0.01^f^	0.03 ± 0.01
Muscle	51 ± 16^c^	30 ± 15	84 ± 5^b,d,f^	24 ± 7
Bone	16 ± 3	11 ± 5	24 ± 10^d,f^	6 ± 2

One-way ANOVA with Bonferroni’s multiple comparisons test was performed to find significant differences. ^a^Significant difference between [^99m^Tc]Tc(CO)_3_-H_6_-G3 and [^99m^Tc]Tc(CO)_3_-G3-H_6_. ^b^Significant difference between [^99m^Tc]Tc(CO)_3_-H_6_-G3 and [^99m^Tc]Tc(CO)_3_-(HE)_3_-G3. ^c^Significant difference between [^99m^Tc]Tc(CO)_3_-H_6_-G3 and [^99m^Tc]Tc(CO)_3_-G3-(HE)_3_. ^d^Significant difference between [^99m^Tc]Tc(CO)_3_-G3-H_6_ and [^99m^Tc]Tc(CO)_3_-(HE)_3_-G3. ^e^Significant difference between [^99m^Tc]Tc(CO)_3_-G3-H_6_ and [^99m^Tc]Tc(CO)_3_-G3-(HE)_3_. ^f^Significant difference between [^99m^Tc]Tc(CO)_3_-(HE)_3_-G3 and [^99m^Tc]Tc(CO)_3_-G3-(HE)_3_.

Experimental gamma-camera imaging confirmed the biodistribution results. The SKOV3 xenografts were visualized using all ^99m^Tc-labeled G3 variants (Fig. 5). However, the background activity was the lowest in the case of [^99m^Tc]Tc(CO)_3_-(HE)_3_-G3. Particularly, the activity accumulation in liver was noticeably lower compared to other variants.

## Discussion

Clinical radionuclide molecular imaging utilizes two major modalities, PET and SPECT. Until very recently, there was a consensus that SPECT is appreciably cheaper and more available, while PET provides better spatial resolution, sensitivity and quantitation accuracy^27^. Accordingly, the focus in development of radiolabeled antibodies and peptides was shifted to the use of positron-emitting radionuclides during the last decade^27–30^. Introduction of new semiconductor cadmium zinc telluride (CZT) detectors for whole body SPECT/CT has changed the situation dramatically. The spatial resolution of CZT-SPECT was improved two-fold compared with conventional Anger-camera based SPECT^31^, which enhanced the sensitivity of imaging due to lower partial volume effect. In addition, the use of CT-based attenuation correction has appreciably improved the quantitation accuracy of CZT-SPECT. The performance of CZT-SPECT in static imaging is approaching the performance of PET, but at appreciably lower price. It makes sense again to develop imaging probes for SPECT. The development of cheap ^99m^Tc-based imaging probes would further promote CZT-SPECT imaging.

The use of histidine-based tags as chelators for [^99m^Tc]Tc(CO)_3_ offers several advantages^25,32^. They simplify purification of recombinant proteins using IMAC. They are incorporated into the protein by genetic engineering, and thus avoid the need for chelator conjugation and purification, which reduces production costs. Furthermore, histidine tags enable site-specific coupling of the radionuclide, which minimizes the risk of undesirable modification of amino acids in the binding site of a scaffold protein and provides a uniform labeled product with reproducible biodistribution properties.

**Figure 2:**
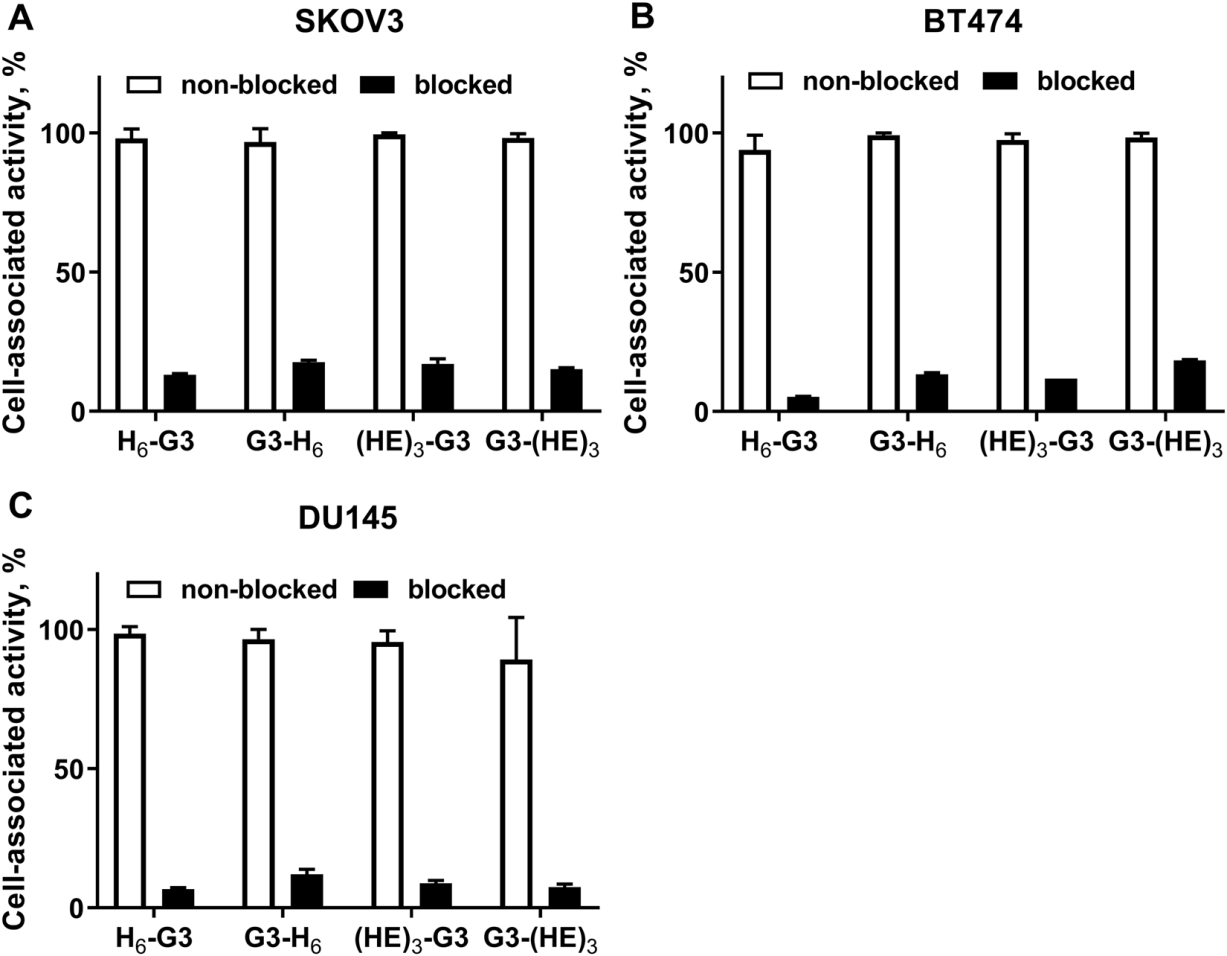
Binding specificity of [^99m^Tc]Tc-labeled G3 variants to SKOV3 (**A**), BT474 (**B**) and DU145 (**C**) HER2-expressing cell lines *in vitro*. Radiolabeled compounds were added at 1 nM concentration; 100-fold molar excess of the corresponding non-labeled G3 variant was added for blocking of HER2 receptors. Data are presented as mean from three samples ± standard deviation (SD).

**Figure 3:**
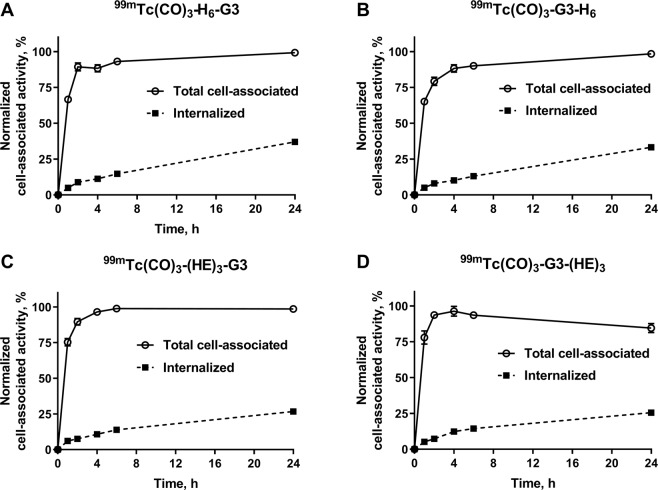
Cellular processing of ^99m^Tc-labeled G3 variants during continuous incubation with HER2-expressing SKOV3 cells over 24 h. Data are presented as mean from three samples ± SD.

**Figure 4:**
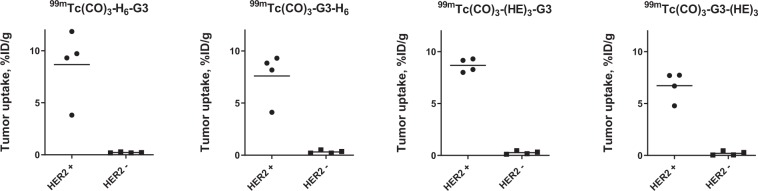
Specificity of HER2-targeting by ^99m^Tc-labeled G3 variants *in vivo*. Tumor uptake in BALB/C nu/nu mice bearing HER2-positive SKOV3 xenografts was compared to the uptake in mice bearing HER2-negative Ramos xenografts for each variant. Data is presented as individual data points from four mice per group.

We have selected the G3 DARPin for optimization in this study, because it demonstrated appreciably better imaging properties with both ^99m^Tc and ^125^I labels compared with 9_29 DARPin^22^. Four G3 variants containing a histidine-containing tag at either N-terminus (H_6_-G3 and (HE)_3_-G3) or C-terminus (G3-H_6_ and G3-(HE)_3_) were generated. Labeling of all variants with [^99m^Tc]Tc(CO)_3_ using CRS kit was quite efficient, stable and provided high radiochemical purity after purification by size-exclusion chromatography (Tables 1, 2). All labeled variants preserved specific binding to HER2-expressing cells *in vitro* (Fig. 2) and had similar affinity to living HER2-expressing cells (Table 3). The cellular processing of all labeled variants had a similar pattern with rather slow internalization (Fig. 3). The internalized activity at 24 h was somewhat higher in the case of hexahistidine-containing variants, which might indicate somewhat stronger residualizing properties in this case. All radiolabeled variants demonstrated much higher accumulation in HER2-positive SKOV3 xenografts than in HER2-negative Ramos xenografts (p < 0.005) (Fig. 4), which clearly demonstrated HER2-specificity of targeting *in vivo*. There was no significant difference between the tumor uptake of different G3 variants at 4 h after injection. The major finding of this study was the difference of uptake in normal tissues (Table 4). Particularly, the uptake of [^99m^Tc]Tc(CO)_3_-(HE)_3_-G3 in a majority of normal tissues was significantly lower compared to the three other variants (Table 5). The influence of the tag composition was more pronounced for placement of the tag at the N-terminus. Accordingly, there was a significant difference in tumor-to-organ ratio between [^99m^Tc]Tc(CO)_3_-(HE)_3_-G3 and other radiolabeled variants (Table 5). Importantly, [^99m^Tc]Tc(CO)_3_-(HE)_3_-G3 provided significantly higher tumor-to-liver, tumor-to-muscle and tumor-to-bone ratios, facilitating contrast at sites where metastases are frequently located. Data for radioiodinated G3^22^ showed that tumor-to-blood, tumor-to-salivary gland, tumor-to-lung and tumor-to-muscle ratios increased at 24 h compared to 6 h, and that imaging next day after injection might increase the imaging contrast. In the case of ^99m^Tc-labeled variants, there was an increase only in tumor-to-blood ratio at the later time point (Table 4). The possible reason for this could be the residualizing nature of [^99m^Tc]Tc(CO)_3_ label, which was then retained in normal tissues after internalization as efficiently as in tumor. Accordingly, the clearance of activity from normal tissues was as slow as from tumors, and the contrast did not increase with time. Thus, the imaging at the day of injection would be optimal for [^99m^Tc]Tc(CO)_3_-(HE)_3_-G3. This matches well with the 6 h physical half-life of ^99m^Tc.

**Figure 5:**
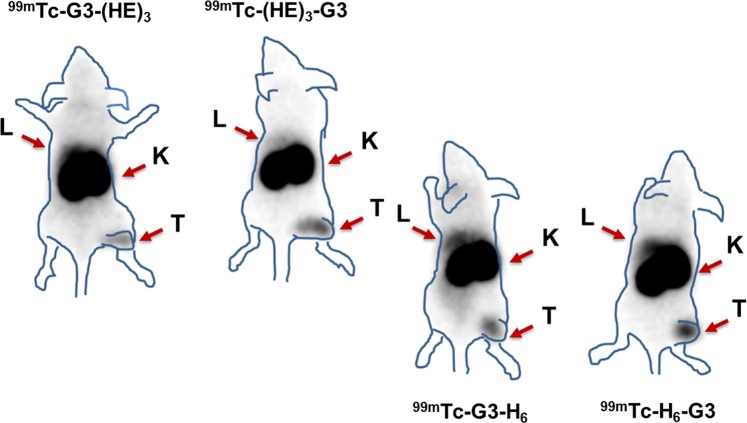
Gamma-camera imaging of BALB/C nu/nu mice bearing SKOV3 xenografts injected with ^99m^Tc-labeled G3 variants (3.2 μg, 8 MBq) at 4 h pi. Contours were derived from a digital photograph and superimposed over images to facilitate interpretation. Arrows are pointing at liver (L), kidneys (K) and tumor (T).

Gamma camera imaging confirmed the *ex vivo* measurements data (Fig. 5) showing that [^99m^Tc]Tc(CO)_3_-(HE)_3_-G3 provided higher tumor-to-background contrast in general and tumor-to-liver in particular.

The fact that physicochemical properties of a radionuclide/chelator complex have a strong influence on biodistribution of radiolabeled peptides is well known^33^. Apparently, an introduction of a chelator and a radionuclide influences charge and lipophilicity of a peptide, which in turn modifies off-target interactions. This affects the predominant excretion pathway, off-target binding to normal tissues and binding to blood proteins resulting in slowing blood clearance rate. Although scaffold proteins are typically bigger than short tumor-targeting peptides, a substantial effect of labeling strategy on biodistribution properties of an ESP affibody molecule has also been shown earlier^32^. A noticeable influence of a radionuclide/chelator combination on uptake in normal tissues was observed for another ESP, ADAPT^34^. Unfortunately, there are no general rules of thumb permitting selection of an optimal label. Probably, the only applicable principle is that the use of more hydrophilic radionuclide/chelator combinations reduces the hepatic uptake of activity^35–37^ and binding to plasma proteins^38,39^. However, there are multiple and unexpected exemptions from these rules. For example, the use of a hydrophilic and negatively charged HEHEHE-tag at the C-terminus of affibody molecules for labeling with [^99m^Tc]Tc(CO)_3_ resulted in a very substantial decrease of hepatic uptake while the use of equally hydrophilic but positively charged HKHKHK-tag in the same position resulted in a substantial increase of hepatic uptake^25^. Furthermore, the same tags provided a different effect that was dependent on their position in affibody molecules^25^.

We found in this study that the influence of tag position and composition on biodistribution of DARPin G3 resembled the influence on biodistribution of affibody molecules^25^, i.e. hydrophilic tags at N-terminus provided the best imaging properties. However, this observation is different from what was previously found for ADAPTs, where the effect of the HEHEHE tag at N-terminus on biodistribution was minor^26^. It has to be noted, though, that the N-terminus of ADAPT6 contains a number of negatively charged amino acids, glutamates and aspartates, which might additionally provide an effect similar to the effect of HEHEHE-tags. Taken together the available data for affibody molecules, ADAPTs and DARPins, we can conclude that the effect of histidine-containing tags depends on local distribution of charge and lipophilicity on a protein’s surface and is determined by neighbor amino acids in a scaffold protein. However, prediction of this effect for every type of ESP is associated with a high degree of uncertainty. Therefore, a systematic investigation of this effect, as presented here, is necessary to ensure that a tracer with optimal properties is produced.

## Conclusion

Both composition and position of histidine-containing tags have a strong influence on biodistribution of anti-HER2 G3 DARPin labeled using [^99m^Tc]Tc(CO)_3_. The [^99m^Tc]Tc(CO)_3_-(HE)_3_-G3 variant provided the highest tumor-to-lung, tumor-to-liver, tumor-to-bone and tumor-to-muscle ratios, which should improve sensitivity of HER2 imaging in metastases. The labeling strategy for molecular imaging probes based on scaffold proteins should be evaluated to provide not only a stable coupling of a radionuclide, but also the most favorable biodistribution pattern.

## Materials and Methods

### Reagents, instruments, statistics

Molecular weight of DARPins was measured by liquid chromatography-electrospray ionization-mass spectrometry (LC-ESI-MS) on a 6520 Accurate Q-TOF LC/ MS (Agilent). Instant thin-layer chromatography (iTLC) analysis was performed using iTLC silica gel strips (Varian, Lake Forest, CA, USA). The radioactivity distribution along strips was measured using a Cyclone storage phosphor system (Packard) and analyzed by OptiQuant image analysis software. Radio-HPLC analysis was performed using Hitachi Chromaster HPLC system with a radioactivity detector. Vydac RP C18 protein column (300 A, 3 × 150 mm, 5 μm particle size) was used for analysis. Solvent A was 0.1% trifluoroacetic acid in water, solvent B was 0.1% trifluoroacetic acid in acetonitrile. Flow rate was 1 mL/min with a gradient of 5% B to 80% B over 20 minutes. Size-exclusion chromatography was performed using NAP-5 columns (GE Healthcare). Radioactivity was measured using an automated gamma-spectrometer with a NaI(TI) detector (1480 Wizard, Wallac, Finland). Technetium-99m was obtained as pertechnetate by elution of Ultra-TechneKow generator (Mallinckrodt) with sterile 0.9% sodium chloride (Mallinckrodt, The Netherlands). The CRS kits for production of tricarbonyl technetium were purchased from the Center for Radiopharmaceutical Sciences (PSI, Villigen, Switzerland). SKOV3, BT474, DU145 and Ramos cells were purchased from the American Type Culture Collection (ATCC) and were cultured in RPMI medium supplemented with 10% fetal bovine serum (FBS), 2 mM L-glutamine, 100 IU/ml penicillin and 100 µg/ml streptomycin in a humidified incubator with 5% CO_2_ at 37 °C, unless stated otherwise.

Statistical analysis of biological data was performed using GraphPad Prism (Windows GraphPad Software, San Diego, CA, USA) to find any significant differences (p < 0.05), when two sets of data were compared. An unpaired two-tailed t test was used for analysis unless stated otherwise. For comparison of three or more sets of data, a one-way ANOVA test with Bonferroni correction for multiple comparisons was applied (Windows GraphPad Software, San Diego, CA, USA).

### Protein production

The proteins G3-H_6_, H_6_-G3, (HE)_3_-G3 and G3-(HE)_3_ were produced in *E*. *coli* strain BL21(DE3) (Novagen-EMD Millipore, Madison, WI 53719, USA). The DARPin G3 gene nucleotide sequence was deduced from a DARPin G3 amino acid sequence deposited in PDB (accession number PDB: 2JAB) taking into account the codon usage in highly expressed *E*. *coli* genes with the help of the freely distributed program DNABuilder (http://www.innovationsinmedicine.org/software/DNABuilder/). The gene was assembled by PCR from chemically synthesized oligonucleotides of 50 bp in length having partially complementary sequences. The additional histidine-containing sequences (tags) were added to the *N*- or *C*-terminus of the DARPin G3 gene by PCR. The amino acid sequence for H_6_-G3 was as follows: MRGSHHHHHHGSDLGKKLLEAARAGQDDEVRILMANGADVNAKDEYGLTPLYLATAHGHLEIVEVLLKNGADVNAVDAIGFTPLHLAAFIGHLEIAEVLLKHGADVNAQDKFGKTAFDISIGNGNEDLAEILQKLN. The amino acid sequence for G3-H_6_ was as follows: MDLGKKLLEAARAGQDDEVRILMANGADVNAKDEYGLTPLYLATAHGHLEIVEVLLKNGADVNAVDAIGFTPLHLAAFIGHLEIAEVLLKHGADVNAQDKFGKTAFDISIGNGNEDLAEILQKLNGSHHHHHH. The amino acid sequence for (HE)_3_-G3 was as follows: MRGSHEHEHEGSDLGKKLLEAARAGQDDEVRILMANGADVNAKDEYGLTPLYLATAHGHLEIVEVLLKNGADVNAVDAIGFTPLHLAAFIGHLEIAEVLLKHGADVNAQDKFGKTAFDISIGNGNEDLAEILQKLN. The amino acid sequence for G3-(HE)_3_ was as follows: DLGKKLLEAARAGQDDEVRILMANGADVNAKDEYGLTPLYLATAHGHLEIVEVLLKNGADVNAVDAIGFTPLHLAAFIGHLEIAEVLLKHGADVNAQDKFGKTAFDISIGNGNEDLAEILQKLNGSHEHEHE.

Expression, isolation and purification was performed according to the methodology described earlier^22^. The genes for G3-H_6_, H_6_-G3 and (HE)_3_-G3 were directly cloned into the plasmid vector pET39b between the restriction sites *Nde*I and *Hin*dIII. The gene for G3-(HE)_3_ was fused to 3′-terminus of the SUMO^40^ gene by overlapping PCR^41^. The junction between two genes encoded the following amino acid sequence *(SUMO)-*QIGG†DLGKK*-(Darpin)*. The 5′-terminus of SUMO gene was extended by the coding sequence of GHHHHHHGS. The hybrid SUMO-DARPin G3-(HE)_3_ gene was cloned into pET39b plasmid vector between restriction sites *Nde*I and *Hin*dIII. Briefly, *E*. *coli* was grown in the autoinduction ZYM-5052 medium^42^ containing 100 μg/mL kanamycin at 25 °C. The cells were harvested by centrifugation and resuspended in lysis buffer (200 mM Tris-HCl, 500 mM sucrose, 1 mM EDTA, pH 8.0, 1 mM PMSF, 60 μg/mL lysozyme). The suspension was diluted two-fold with distilled water and incubated at room temperature for 30 min. Cells were broken on ice by a Vibra Cell ultrasonic liquid processor VCX130 (Sonics, USA). After addition of imidazole (30 mM) and NaCl (500 mM), the cellular debris were pelleted at 70000 g at 4 °C for 30 min. The supernatant was filtered through a 0.22 μm membrane and applied on a HisTrap HP (1 mL) column (GE Healthcare, USA) equilibrated with 20 mM NaPi, pH 7.5, 500 mM NaCl, 30 mM imidazole. The bound proteins were eluted with a linear 30–500 mM imidazole gradient. The fractions were analyzed by 15% SDS-PAGE. In the case of DARPin (HE)_3_-G3 the imidazole in the cellular lysate, in the column equilibration and wash buffers was omitted to prevent blocking of protein binding to the Ni-chelating sorbent. The homemade SUMO hydrolase (ULP1)^40^ was added to the combined fractions containing SUMO-DARPin G3-(HE)_3_ at the molar ratio 1:100 (enzyme: substrate). After overnight incubation at 6 °C, the reaction mixture was diluted three-fold with 20 mM NaPi, pH 7.5, and applied to a HisTrap HP (1 mL) column equilibrated with 20 mM NaPi, pH 7.5, 30 mM imidazole. The flow-through eluate was collected.

Solutions containing DARPins were diluted with 25 mM Tris-HCl, pH 8.0 and loaded on a MonoQ 10/100 GL column (GE Healthcare, USA) equilibrated with 25 mM Tris-HCl, pH 8.0. The bound proteins were eluted with a linear 0–1 M NaCl gradient. The fractions containing DARPins were pooled, concentrated with Amicon Ultra-15 centrifugal filter, and sterilized by filtration through 0.22 μm membrane. Protein concentrations were determined by UV spectroscopy using ε_280_ = 2560 M^−1^ cm^−1^.

### Radiolabeling of G3 variants

Site-specific radiolabeling of histidine tag-containing G3 variants with [[^99m^Tc]Tc(CO)_3_]^+^ was performed as described earlier by Deyev *et al.*^22^. Briefly, the eluate (500 μL) containing ca. 3–5 GBq of [^99m^Tc]Tc was added to a CRS kit, which was then incubated at 100 °C for 30 min. After incubation, 12 μL from the CRS reaction mixture was added to 40 μg (2.75 nmol) of a G3 variant in 33 μL of PBS. The resulting mixture was incubated for 60 min at 60 °C. Then, a 5000-fold molar excess of histidine (13.7 μmol, 212 μL of 10 mg/mL in PBS) was added to the mixture and further incubated for 15 min at 60 °C. Radiolabeled G3 variants were purified using NAP-5 columns pre-equilibrated and eluted with PBS. Radiochemical yield and purity were measured using radio-iTLC in PBS^24^. The radiolabeled DARPins and the reduced- hydrolyzed technetium (RHT) colloid remained at the application point, while [^99m^Tc]TcO_4_^−^, [[^99m^Tc]Tc(CO)_3_]^+^ and its complex with histidine migrated with the solvent front. To determine the presence of RHT, iTLC strips were eluted with pyridine:acetic acid:water (10:3:1.5)^24^. In this system, the RHT colloid stayed at the application point, while radiolabeled DARPins, [^99m^Tc]TcO_4_^−^, [[^99m^Tc]Tc(CO)_3_]^+^ and its complex with histidine migrated with the solvent front.

Stability test was performed by incubating the purified radiolabeled proteins with 5000-fold molar excess of histidine in PBS for up to 3 h at room temperature; control samples were incubated in PBS. Samples were analyzed by radio-iTLC analysis in PBS^22^.

### *In vitro* studies

The *in vitro* studies were performed as described previously^22^. Cells (ca. 1 × 10^6^ cells/ dish) were seeded one or two days before the experiment, three dishes per group.

Binding specificity of ^99m^Tc-labeled G3 variants to HER2 was evaluated using HER2-expressing SKOV3, BT474 and DU145 cells. To saturate HER2 receptors, a 100-fold excess of non-labeled G3 variant (100 nM) was added to the control group of cells, medium only was added to the second group. After 30 min, ^99m^Tc-labeled G3 variants were added to both groups at 1 nM concentrations. After incubation for 1 h at 37 °C, the cell medium was collected, cells were washed with 1 mL of fresh medium and 1 mL of 1 M NaOH was added to lyse the cells. The cell lysate was collected after 30 min of incubation with NaOH. The radioactivity in each fraction was measured to calculate the percent of cell-bound radioactivity, which was normalized to the maximum uptake value in each dataset.

### Affinity measurement using LigandTracer

The binding kinetics of ^99m^Tc-labeled G3 variants to living SKOV3 cells was measured using LigandTracer (Ridgeview Instruments AB, Vänge, Sweden), as described earlier^22^. Kinetics was recorded at room temperature in real time. Increasing concentrations of radiolabeled G3 variants (0.5 and 2 nM) were added to cells followed by replacement of media and measurements of retention in the dissociation phase. The TraceDrawer Software (Ridgeview Instruments AB, Vänge, Sweden) was used to calculate the dissociation constants based on association and dissociation rates.

Cellular processing of ^99m^Tc-labeled G3 variants by SKOV3 cells was studied during continuous incubation by an acid-wash method^22^. Radiolabeled DARPins (1 nM) were added to cells and incubated at 37 °C in a humidified incubator. At 1, 2, 4, 6 and 24 h post-addition, the media was collected from one set of dishes and cells were washed once with serum-free media (1 mL). To collect the membrane-bound DARPins, the cells were treated with 0.2 M glycine buffer containing 4 M urea, pH 2.0 (1 mL) on ice for 5 min, the buffer was collected, and the cells were washed once with the same buffer (1 mL). To collect the internalized DARPins, the cells were treated with 1 M NaOH (1 mL) for 30 min. The activity in every fraction was measured. The activity in the acid fractions was considered as bound to cell membrane, in the alkaline fractions- as internalized. The data was normalized to the maximum value of cell-bound activity in each dataset.

### Animal studies

Animal studies were performed according to national legislation on laboratory animal protection and were approved by the Ethical Committee for Animal Research in Uppsala. For tumor implantation, 10^7^ of HER2-expressing SKOV3 cells or 5 × 10^6^ of HER2-negative Ramos cells in 100 µL of media were subcutaneously injected in the right hind leg of female BALB/c nu/nu mice. The experiments were performed three weeks after implantation. The average animal weight was 18 ± 1 g in the SKOV3 groups, 18 ± 1 g in the Ramos group. The average tumor weight was 0.06 ± 0.02 g for SKOV3 xenografts and 0.2 ± 0.1 g for Ramos xenografts. For biodistribution studies, mice were intravenously (i.v.) injected with ^99m^Tc-labeled G3 variant (0.22 nmol, 3.2 μg) in 100 μL of 1% BSA in PBS/mouse (30 kBq for ^99m^Tc-labeled DARPins for 4 h groups and 320 kBq for 24 h groups). The injected amount of protein was adjusted to by a corresponding non-labeled G3 variant. At 4 and 24 h post-injection (pi) mice were anesthetized by an intraperitoneal injection of ketamine and xylazine solution and sacrificed by heart puncture. Blood was collected with a heparinized syringe, organs were collected, weighed and activity was measured using a gamma spectrometer. The percent of injected dose per gram of sample (%ID/g) was calculated, except for gastrointestinal tract and carcass where %ID per whole sample was calculated.

For imaging mice bearing SKOV3 xenografts were injected with ^99m^Tc-labeled G3 variants (3.2 µg, 8 MBq) in 100 μL of 1% BSA in PBS/mouse and sacrificed by CO_2_ inhalation at 4 h pi. The imaging was performed using an Infinia gamma-camera (GE Healthcare) equipped with a low-energy high resolution collimator. Static images (30 min) were obtained in a 256 × 256 matrix.

## Supplementary information

Supplementary Information
