# Supplementary material for: Optimal composition and position of histidine-containing tags improves biodistribution of 99mTc-labeled DARPin G3
Source: Sci Rep. 2019 Jun 28;9:9405. doi: 10.1038/s41598-019-45795-8 (PMC6599047; doi:10.1038/s41598-019-45795-8)
Supplement: Supplementary file 1 — Supplementary Information [file 41598_2019_45795_MOESM1_ESM.pdf]

Supplementary Information

Optimal composition and position of histidine-containing tags improves biodistribution of ^{99m}Tc-labeled DARPin G3

Anzhelika Vorobyeva, Alexey Schulga, Elena Konovalova, Rezan Güler, John Löfblom, Mattias Sandström, Javad Garousi, Vladimir Chernov, Olga Bragina, Anna Orlova, Vladimir Tolmachev, Sergey M. Deyev,

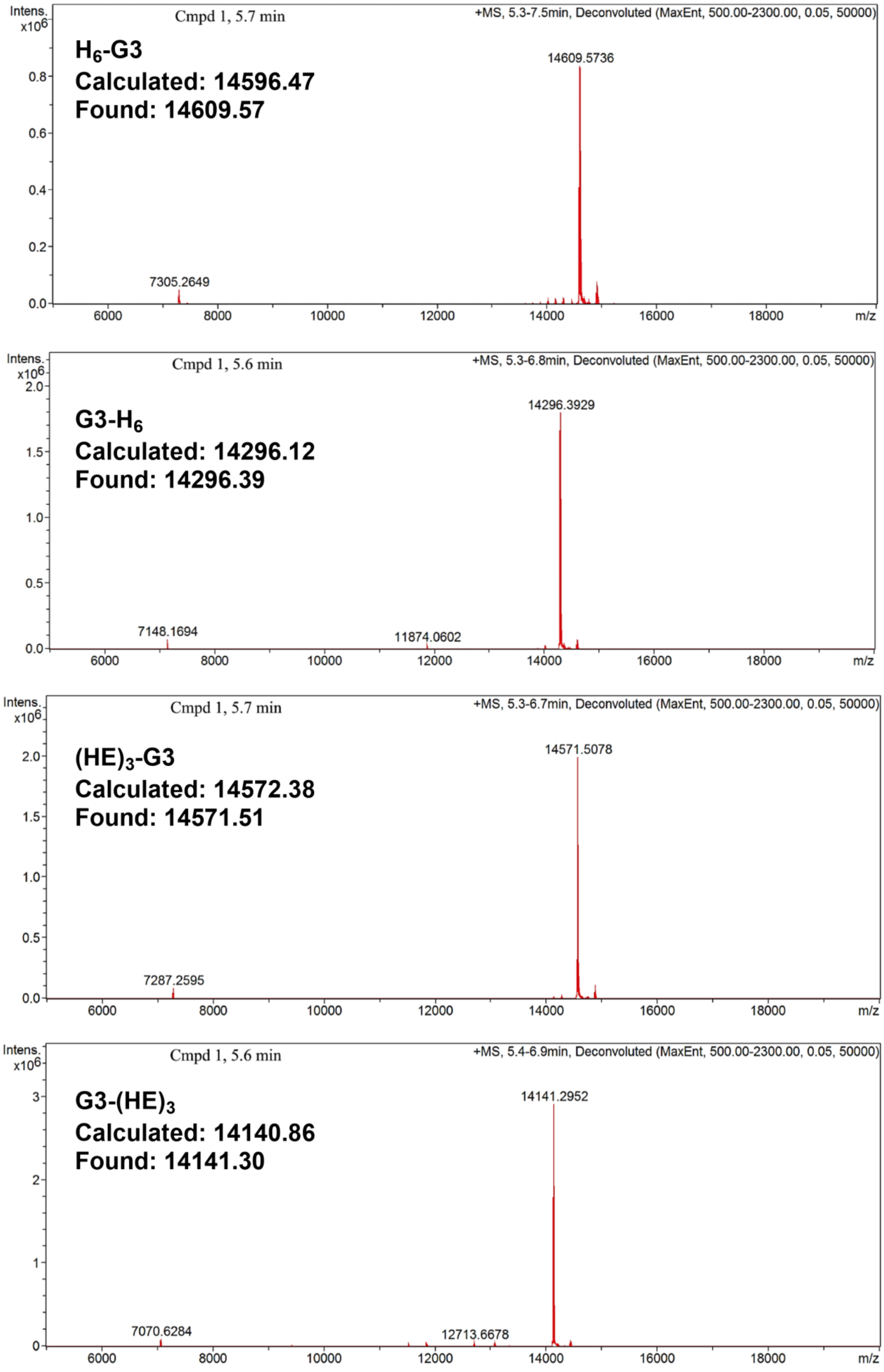

SI Figure 1. ESI-MS analysis of G3 variants.

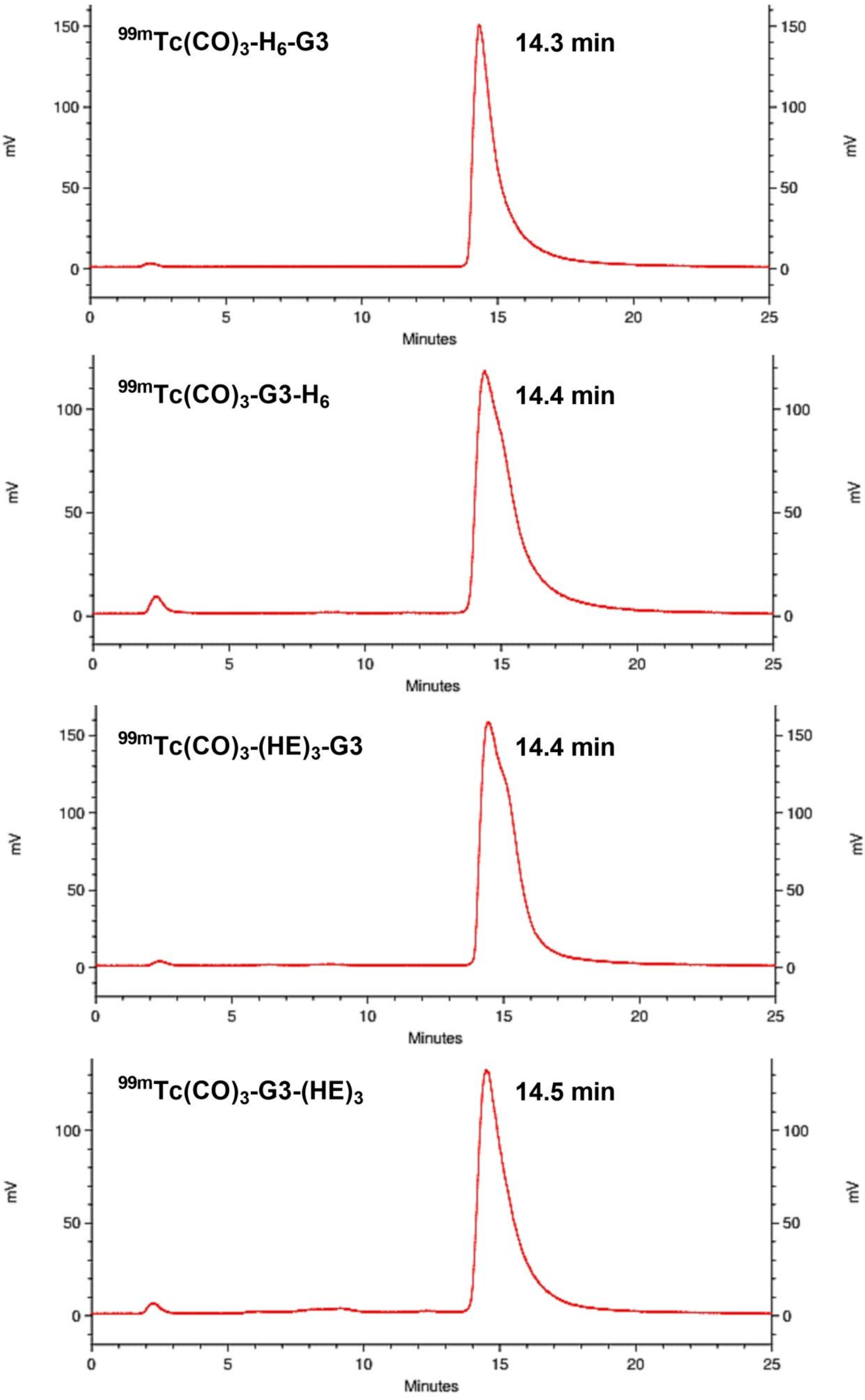

SI Figure 2. Radio-HPLC analysis of ^{99m}Tc-labeled G3 variants. Retention time of the major peak (in mins) is shown.
